# Solvent polarity effects on the FTIR spectrum, and thermodynamic and electronic properties of metronidazole and its binding with antibacterial drugs: a DFT and molecular docking study

**DOI:** 10.1039/d5ra02359a

**Published:** 2025-08-11

**Authors:** Desta Regassa Golj, Megersa Olumana Dinka, Umer Sherefedin, Abebe Belay, Dereje Gelanu, Gadisa Deme Megersa

**Affiliations:** a Department of Civil Engineering Science, Faculty of Engineering and the Built Environment, University of Johannesburg Johannesburg 2006 South Africa; b Department of Physics, College of Natural and Computational Sciences, Madda Walabu University Bale Robe, P.O. Box 247 Ethiopia umerphysics2005@gmail.com; c Department of Applied Physics, School of Applied Natural Sciences, Adama Science and Technology University Adama, P.O. Box 1888 Ethiopia

## Abstract

Metronidazole is widely used as an antimicrobial, particularly effective against anaerobic bacteria and protozoan infections. This study investigates solvent polarity effects on the Fourier transform infrared (FTIR) spectrum, and thermodynamic and electronic properties of metronidazole *via* semiempirical, Hartree–Fock (HF), and density functional theory (DFT) methods. Its binding with antibacterial drugs was also investigated *via* molecular docking. The results showed that in water, the dipole moment and polarizability increased, indicating enhanced solubility and reactivity. Solvent-induced changes in bond lengths and angles are important for understanding the behavior of metronidazole in biological systems. FTIR reveals changes in molecular interactions due to solvation effects, especially hydrogen bonding in water. Thermodynamic calculations further revealed that polar solvents increase the energy and dipole moment, enhancing the reactivity of the molecule. Frontier molecular orbital (FMO) analysis indicated that the molecules are more stable in polar environments, while UV-Vis spectral shifts showed that the solvent affects the electronic properties. Molecular docking studies with antibacterial proteins revealed that metronidazole binds strongly to proteins, with the metronidazole-4kov complex showing the highest binding affinity. Molecular docking of metronidazole with secnidazole, tizoxanide, and caffeine enhances the binding affinities, suggesting synergistic effects. In conclusion, this study emphasizes the importance of solvent polarity for optimizing the antibacterial properties of metronidazole and its molecular docking with other drugs.

## Abstract 2

This study investigates solvent polarity effects on the FTIR spectrum, and thermodynamic and electronic properties of metronidazole via semiempirical, Hartree-Fock, and DFT methods. Its binding with antibacterial drugs was also investigated.

## Graphical abstract

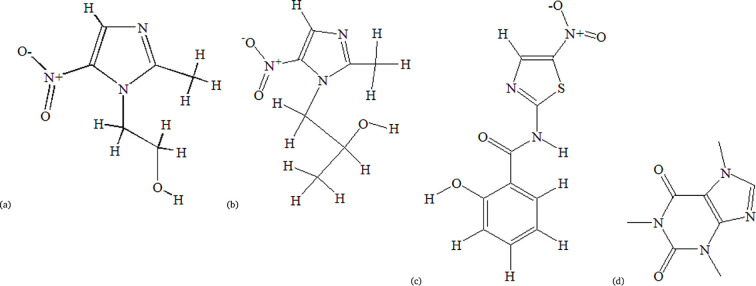

## 1 Introduction

**Fig. 1:**
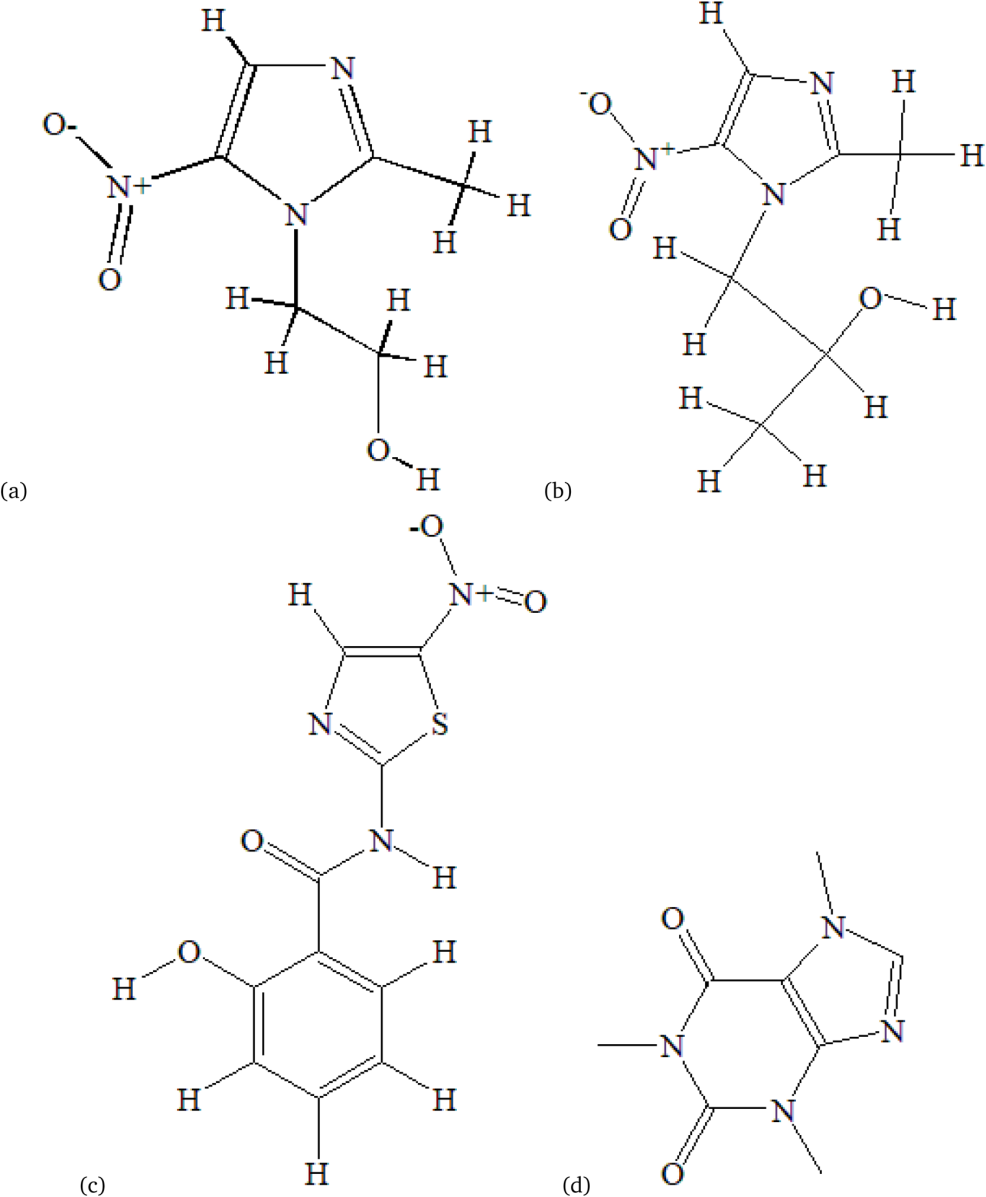
The chemical structures of metronidazole (a), secnidazole (b), tizoxanide (c), and caffeine (d).

Metronidazole (MNZ) is an antimicrobial drug used to treat infections caused by anaerobic bacteria and protozoa.^1^Fig. 1(a) shows the molecular structure of metronidazole. Its activity is closely related to its chemical structure, especially the nitro group at position 5 of the imidazole ring. This nitro group is essential for its function.^2,3^ Under anaerobic conditions, bacterial and protozoal enzymes reduce the nitro group, creating reactive intermediates. These intermediates then interact with microbial DNA, causing strand breaks and structural damage. This disrupts DNA replication and nucleic acid synthesis, leading to cell death.^4^ A previous study revealed that metronidazole nanoemulsions have strong antibacterial activity against both Gram-positive and Gram-negative bacteria. The particles were uniform and well dispersed.^5^ In another report, metronidazole-nanosuspension-loaded dissolving microarray patches improved skin penetration. These patches effectively treat skin and soft tissue infections caused by *Bacteroides fragilis*.^6^ Changes in drug structure can alter drug function and have recently gained attention because of the interaction of functional groups with environmental factors, such as solvents, that affect drug activity. These groups control the strength and nature of interactions with solvents and other drugs.^7^ Consequently, investigating the interactions between solvents and drugs is essential for understanding biological processes. It also provides valuable insights into the changes in electronic distribution that occur upon excitation.^8^

Recently, the effects of solvent polarity on the dipole moment, Fourier transform infrared (FTIR) spectrum, highest occupied molecular orbital (HOMO), lowest unoccupied molecular orbital (LUMO), chemical reactivity, density of states (DOS), electrostatic potentials (ESPs), ultraviolet-visible (UV-Vis) spectra, and fluorescence of various drugs have been studied. These drugs include metformin hydrochloride,^9^ aspirin,^10^ zaleplon,^11^ nifenazone,^12^ imiquimod,^13^ sulfisoxazole,^14^ and hydroxycinnamic acids like sinapic acid,^15^ chlorogenic acid and caffeic acid,^16^ and ferulic acid.^17^ The results of these studies revealed the general solvent effect, which is related to the relative permittivity and refractive index. In addition, a specific effect, driven by hydrogen bonding and intermolecular charge transfer, occurred between the drugs and solvents. As the polarity of solvents changes, shifts in the absorption and emission peaks occur, leading to changes in the dipole moment, FTIR spectrum, HOMO–LUMO gap, chemical reactivity, DOS, and MEP of selected drugs due to solvent–drug interactions. Estimating these changes in drug properties in both the ground and excited states through solvatochromic effects *via* density functional theory (DFT) and time-dependent DFT (TD-DFT) is essential for understanding the electronic properties and structural modifications of these drugs. The biological activities of molecules depend on their molecular structure. Even small changes in drug properties due to solvent–drug interactions can signal structural modifications. These modifications can, in turn, affect the biological activities of the drug, making properties such as the dipole moment, FTIR spectrum, HOMO–LUMO gap, chemical reactivity, DOS, and MEP important measurable factors in drug analysis.^9–17^

On the other hand, the binding between ligands and proteins,^18^ multiple ligand–protein interactions,^19,20^ or ligand–ligand interactions^21^ are crucial for biological activity. The pharmacological effectiveness of a drug largely depends on its ability to bind with proteins or its potential for drug–drug interactions.^22^ Any changes in this binding can directly impact the drug’s activity. Recently, the simultaneous use of multiple drugs has increased, both knowingly and unknowingly.^23^ This concurrent drug use can lead to interactions that may either enhance or diminish the biological activity of a drug, with such interactions occurring between proteins and ligands or through multiple ligands interacting with proteins.^24^ Sherefedin *et al.* (2025) reported the molecular docking of hydroxycinnamic acids such as ferulic, *p*-coumaric, caffeic, and sinapic acids with anticancer-related proteins such as 3M18, 5EKN, and 6YKY.^25^ The results revealed strong binding affinities, with favorable root mean square deviation (RMSD) values, indicating stable interactions and potential as anticancer agents. Molecular docking studies were also performed on salicylidene–aniline and their metal mixed-ligand complexes in interaction with caffeine. The results showed that the metal–caffeine complexes had stronger binding affinities than the free ligands, suggesting enhanced biological potential.^26^ Another study examined the impact of caffeine and flavonoids on tigecycline’s binding to human serum albumin. Docking results revealed that both compounds altered tigecycline’s binding affinity.^27^ Woldegiorges *et al.* (2022) reported that the interaction of caffeine with levofloxacin and norfloxacin leads to significant fluorescence quenching, indicating strong molecular interactions between caffeine and these drugs. The quenching effect is attributed to the binding of caffeine with the fluorophores of these drugs, which alters their photophysical properties.^28^ Furthermore, the interaction between caffeine and aspirin in Kopi Balur 1 was investigated. The results showed that this interaction influences the biological activity of the compound.^29^

Previously, research has investigated the effects of solvent polarity on drugs such as metformin, aspirin, zaleplon, and hydroxycinnamic acids, including ferulic, *p*-coumaric, caffeic, and sinapic acids, with a focus on their structure, thermodynamics, and electronic properties *via* DFT and molecular docking methods. However, the impact of solvent polarity on metronidazole, particularly its antibacterial activity, has not been explored. On the other hand, previously, drug–protein and drug–drug interactions have been investigated for other compounds using techniques like molecular docking and fluorescence quenching; however, there is a notable absence of studies specifically examining metronidazole’s interactions with antibacterial proteins or its behavior in multiple-ligand interactions with agents such as secnidazole (Fig. 1(b)), tizoxanide (Fig. 1(c)), and caffeine (Fig. 1(d)). Therefore, this study addresses these gaps by investigating how solvent polarity affects the structure and properties of metronidazole *via* semiempirical, Hartree–Fock (HF), and DFT (B3LYP) methods with various basis sets. It also explores drug–drug interactions, particularly with amino acids, through molecular docking (AutoDock Vina 1.1.2, PyRx version 0.8). The goal is to better understand how solvent polarity and drug interactions influence the biological activity and effectiveness of metronidazole.

## 2 Materials and methods

### 2.1 Tools

ChemDraw Ultra 8 (ref. 30) was used to draw the chemical structures of the ligand and Chem3D Ultra 8 (ref. 31) was used to generate 3D molecular structures of the ligand. GaussView 6 (ref. 32) was utilized for molecular structure building, simulation setup, and visualization. GaussSum^33^ was used to plot the density of states (DOS) analysis. Gaussian 09W^34^ was employed for all density functional theory (DFT) calculations. Chemcraft 1.8 (ref. 35) was employed to interpret and visualize the Gaussian output files. Discovery Studio 2021 (ref. 36) was used for protein preparation and molecular interaction analysis. AutoDock Vina 1.1.2 (ref. 37) was used to predict the binding affinities and docking poses, whereas PyRx version 0.8 (ref. 38) streamlined the virtual screening and docking studies. PyMOL^39^ enabled 3D molecular structure visualization, and Open Babel^40^ was used for chemical file format conversion. The Research Collaboratory for Structural Bioinformatics (RCSB) Protein Data Bank (PDB)^41^ provides the protein structure data, while PubChem provides the ligand data.

### 2.2 Methods

#### 2.2.1 DFT study

**Fig. 2:**
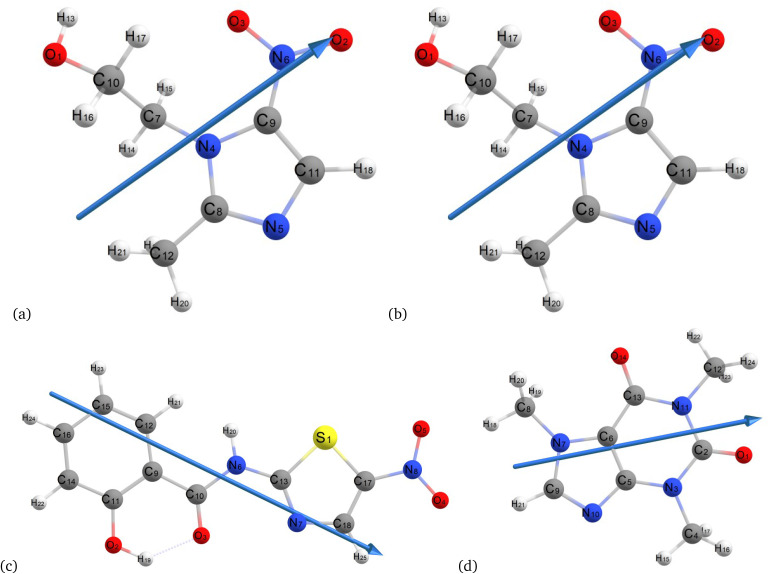
Optimized molecular structures of metronidazole (a), secnidazole (b), tizoxanide (c), and caffeine (d).

Geometric optimizations of metronidazole (Fig. 2(a)) were performed *via* empirical methods using the ZDO basis set with the calculation methods PM6, PDDG, AM1, PM3, and PM3MM, and Hartree Fock and DFT (B3LYP) with various basis sets, including STO-3G*, SDD, 3-21+G*, Aug-CC-pVDZ, 6-31++G (d, p), LANL2DZ, 6-31++G’ (d, p), and 6-311++G (d, p). All computations were performed *via* Gaussian 09 software^34^ for both the vacuum and solvent phases. To assess solvation effects, an integral equation formalism polarizable continuum model (IEFPCM) was utilized.^42^ The optimized geometries were subjected to vibrational analysis to confirm the absence of imaginary vibrations. Following optimization, the geometry parameters (bond lengths, bond angles, and dihedral angles), infrared spectra, HOMO/LUMO levels, density of states, chemical reactivity, and thermodynamic parameters of MNZ were calculated *via* DFT (B3LYP) with the 6-311++G (d, p) basis set. Additionally, the absorption spectra of MNZ were computed *via* time-dependent DFT (TD-DFT) based on the optimized ground-state geometry obtained from DFT.

#### 2.2.2 Molecular docking study

Protein preparation: Protein preparation involved retrieving the 3D structures of target proteins (8fb0, 4kov, 5j62, and 3q5p), which are associated with cancer cell growth and progression, from the Protein Data Bank (http://www.rcsb.org/pdb/). The structures are processed *via* Biovia Discovery Studio,^36^ polar hydrogen is added, and water molecules and heteroatoms are removed to avoid unintended interactions during the docking process. Among the various ligand poses within the protein crystal structure, a specific pose was selected on the basis of its *X*, *Y*, and *Z* coordinates to evaluate binding affinity. The resulting 3D structures were saved in .pdb format.

Ligand preparation: The ligands metronidazole, secnidazole, tizoxanide, and caffeine were prepared *via* ChemDraw Ultra 8.0.^30^ Chem3D Ultra^43^ was used to minimize energy, stabilize their conformations and reduce steric strain. DFT (B3LYP/6-311++G(d,p)) was used to optimize the geometries (Fig. 2(a)–(d)) and was used for docking.

Molecular docking: Molecular docking was performed by importing the cleaned proteins (8fb0, 4kov, 5j62, and 3q5p) into AutoDock Vina 1.1.2.^37^ Kollman and Gasteiger charges were assigned to optimize the electrostatic properties, and AD4 atom types were applied for compatibility with the docking algorithm. The metronidazole ligand was imported, and a torsion tree was added. The active sites for each protein were identified based on the positions of co-crystallized ligands in the crystal structures available from the Protein Data Bank. These positions were used to set the grid box coordinates (*x*, *y*, *z*) in AutoDock Vina 1.1.2, ensuring docking within the biologically relevant binding pockets. After loading the ligand (ligand.pdbqt) and setting the docking parameters, the results were obtained *via* the command prompt, which predicts the binding affinities and amino acid interactions. Post-docking analysis was conducted *via* BIOVIA Discovery Studio, which visualized the binding sites, hydrogen bonds, hydrophobic interactions, and bond distances. The best ligand pose was selected on the basis of hydrogen bond interactions and visualized in both 2D and 3D. In addition, PyRx version 0.8 (ref. 38) was used to dock multiple inhibitors, including metronidazole, secnidazole, and tizoxanide, with the receptor proteins. PyRx version 0.8 (ref. 38) automated the ligand and protein preparation, converting ligands to the PDBQT format for compatibility with AutoDock Vina 1.1.2.^37^ The docking grid was adjusted to target the receptor’s active site, enabling an efficient search for optimal ligand–receptor interactions. Using the Vina algorithm, PyRx version 0.8 (ref. 38) was used to calculate binding affinities and ranked ligand poses based on docking scores. The results were then analyzed to identify the strongest binding conformations. Finally, Discovery Studio was used to visualize binding interactions, focusing on hydrogen bonding, hydrophobic contacts, and key amino acid interactions.

## 3 Results and discussion

### 3.1 Optimizing the chemical structure

The choice of basis set is crucial in computational chemistry and significantly influences the accuracy of the predicted molecular parameters and properties.^44,45^Table 1 presents the dipole moment, polarizability, and thermodynamic properties of metronidazole, evaluated with various semi-empirical methods (PM6, PDDG, AM1, PM3, and PM3MM) using the ZDO basis set in both vacuum and water environments. The dipole moment values indicate that metronidazole has a relatively high polarity in water, with the PM6 method yielding the highest dipole moment of 5.650 D, suggesting enhanced solvation properties and interaction potential in aqueous environments. Polarizability increases significantly in the water phase for most methods, indicating a greater interaction with the solvent, which can positively affect the compound’s solubility and reactivity. Furthermore, variations in thermal energy and heat capacity highlight solvent influences, with water generally leading to more stable properties for metronidazole.

**Table 1:** The calculated dipole moment (*μ*, D), polarizability (*α*, a.u.), thermal energy (*E*, kcal mol^−1^), heat capacity (*C*_V_, cal mol^−1^ K^−1^), and entropy (*S*, cal mol^−1^ K^−1^) of metronidazole *via* semiempirical methods using the ZDO basis set

Calculation method	Vacuum					Water				
	*μ*	*α*	*E*	*C*_V_	*S*	*μ*	*α*	*E*	*C*_V_	*S*
PM6	4.037	83.538	100.854	42.832	112.334	5.650	110.354	100.276	42.998	111.484
PDDG	4.057	76.664	106.825	41.653	109.003	5.164	96.883	106.649	41.621	107.701
AM1	3.704	84.447	111.878	40.083	108.719	4.910	110.895	111.594	40.148	108.305
PM3	3.927	76.897	108.298	42.231	110.403	5.1195	97.412	108.113	42.270	110.229
PM3MM	3.927	76.897	108.298	42.231	110.403	5.119	97.412	108.113	42.270	110.229

Table 2 shows dipole moment, polarizability, thermal energy, heat capacity, and entropy of metronidazole calculated using the HF method. The dipole moment increases notably in water compared to in a vacuum, reflecting the solvent’s polarizing effect, with values ranging from 2.707 D (STO-3G*) in vacuum to 5.696 D (3-21+G*) in water. The polarizability also increases consistently in aqueous medium, indicating enhanced electron cloud distortion. While thermal energy, heat capacity, and entropy show minor fluctuations across basis sets, they remain relatively stable between vacuum and solvent conditions, suggesting that solvation has a more pronounced effect on electrostatic properties than on thermal behavior. Among the tested basis sets, larger and more diffuse functions like Aug-CC-pVDZ and 6-311+G(d,p) yield higher accuracy and consistent values, making them more reliable for capturing solvation effects and molecular response properties.

**Table 2:** The calculated dipole moment (*μ*, D), polarizability (*α*, a.u.), thermal energy (*E*, kcal^−1^ mol^−1^), heat capacity (*C*_V_, cal^−1^ mol^−1^ K^−1^), and entropy (*S*, cal^−1^ mol^−1^ K^−1^) of metronidazole using various basis sets with the HF calculation method

Basis sets	HF (vacuum)					HF (water)				
	*μ*	*α*	*E*	*C*_V_	*S*	*μ*	*α*	*E*	*C*_V_	*S*
STO-3G*	2.707	54.102	125.006	38.764	106.853	3.324	63.322	124.963	38.767	106.898
3-21+G*	3.851	95.916	115.572	39.335	104.145	5.696	130.193	115.211	39.474	103.745
6-31+G (d, p)	3.424	94.989	117.537	38.772	104.422	4.613	125.056	117.247	38.882	104.719
6-311+G (d, p)	3.481	95.51	117.088	38.882	104.706	4.681	125.777	116.806	38.969	104.865
Aug-CC-pVDZ	3.504	100.957	116.995	38.845	104.528	4.772	134.494	116.726	38.928	104.547
LANL2DZ	3.661	87.644	117.272	38.982	104.775	5.066	114.889	117.037	39.002	104.388
SDD	3.658	87.679	117.243	38.985	104.773	5.063	114.93	117.009	39.007	104.398

Table 3, shows the calculated dipole moment (*μ*), polarizability (*α*), thermal energy (*E*), heat capacity (*C*_V_), and entropy (*S*) of metronidazole in vacuum and water using various DFT basis sets. The dipole moment increases in water for all basis sets, indicating stronger molecular polarity due to solvent effects. Smaller basis sets like STO-3G* underestimate *μ* and *α* values, while larger, more flexible basis sets such as 3-21+G* and 6-31+G(d,p), provide higher and more accurate values. Polarizability also rises significantly in water, reflecting enhanced electron cloud distortion. Thermal energy (*E*), heat capacity (*C*_V_), and entropy (*S*) exhibit only slight variations across different basis sets and solvation conditions, suggesting that these thermodynamic properties are relatively insensitive to the level of basis set used in DFT calculations. These findings highlight that selecting an appropriate basis set is critical for accurately capturing electronic properties, especially dipole moment and polarizability, in DFT calculations involving solvation effects.

**Table 3:** The calculated dipole moment (*μ*, D), polarizability (*α*, a.u.), thermal energy (*E*, kcal mol^−1^), heat capacity (*C*_V_, cal mol^−1^ K^−1^), and entropy (*S*, cal mol^−1^ K^−1^) of metronidazole in a vacuum and water

Basis sets	DFT (Vacuum)					DFT (Water)				
	*μ*	*α*	*E*	*C*_V_	*S*	*μ*	*α*	*E*	*C*_V_	*S*
STO-3G*	2.06	61.476	114.017	41.944	109.469	2.632	72.815	114.028	41.998	109.677
3-21+G*	3.944	108.798	108.405	42.132	107.241	5.711	149.643	108.391	42.079	106.526
6-31+G (d, p)	3.611	108.664	109.701	41.655	107.509	5.065	148.19	109.597	41.697	107.497
6-311++G (d, p)	3.439	98.298	109.284	41.66	106.975	5.035	148.392	109.236	41.781	107.913
LanL2DZ	3.737	96.83	109.443	41.808	107.612	5.185	129.154	109.383	41.863	107.836
SDD	3.733	96.977	109.43	41.819	107.621	5.18	129.393	109.369	41.874	107.852

**Fig. 3:**
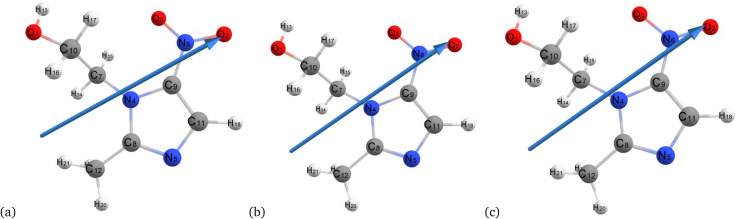
Optimized structures of metronidazole *via* the vacuum semiempirical method (MP6) (a), Hartree–Fock (b), and B3LYP/6-311++G (d, p) (c).

**Fig. 4:**
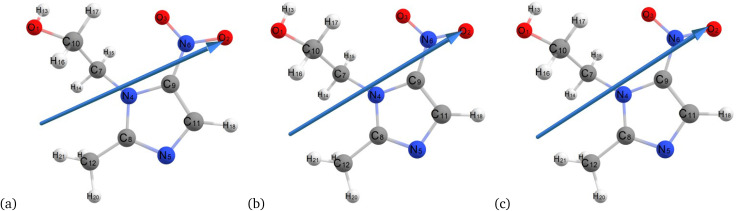
Optimized structures of metronidazole in the water *via* the semiempirical method (MP6) (a), Hartree–Fock (b), and B3LYP/6-311++G (d, p) (c).

Fig. 3 and 4 illustrate the optimized ground-state chemical structures of metronidazole from different computational methods and environments, facilitating a comparative analysis of molecular geometry under vacuum and aqueous conditions. The optimized structures of metronidazole in various environments highlight significant molecular interactions. In Fig. 3, the hydrogen atom H17 interacts with oxygen (O1) and nitrogen atoms, showcasing potential hydrogen bonding interactions. In the absence of solvent, the molecule exhibits a more planar configuration, particularly for the nitro group, with C–N–O angles remaining consistent. However, this stability is sensitive to environmental changes, as evidenced by the planar nature of the nitro group, which suggests an ideal electronic distribution for bonding interactions.

In Fig. 4, the interaction of H17 with the O1 and N atoms is affected. This results in slight shifts and indications of solvent-induced stabilization through additional hydrogen bonding. The nitro group attached to C9 exhibits a discernible out-of-plane twist. This twist is indicative of solvation effects that alter the electron distribution and bond angles compared to its vacuum state. The rotation of the hydroxyl group connected to C10 and the reorientation of the methyl group on C8 reveal the influence of solvent interactions. These interactions affect torsional strain and dielectric stabilization. Furthermore, slight changes in dihedral angles across the molecule suggest that electrostatic forces promote bent conformations in polar environments. This confirms the dynamic response of metronidazole to solvent polarity.

Table 4 provides the optimized geometric parameters of metronidazole in a vacuum at 298.15 K, highlighting the impact of the solvent on the molecular structure. In a vacuum, the bond lengths show typical covalent characteristics, such as O(1)–C(10) at 1.453 Å, while the bond angles exhibit values like C(10)–O(1)–H(13) at 110.355°, reflecting a stable configuration influenced by electronic repulsions. The dihedral angles reveal substantial flexibility, as seen with H(13)–O(1)–C(10)–C(7) at −83.159°, indicating that steric and torsional dynamics could allow the molecule to adopt multiple conformations.

**Table 4:** Calculated optimized parameters of metronidazole in a vacuum at 298.15 K

Bond length	Values (Å)	Bond angles	Values (°)	Dihedral angles	Values (°)
O(1)–C(10)	1.453	C(10)–O(1)–H(13)	110.355	H(13)–O(1)–C(10)–C(7)	−83.159
O(1)–H(13)	0.973	C(7)–N(4)–C(8)	125.536	H(13)–O(1)–C(10)–H(16)	157.693
O(2)–N(6)	1.271	C(7)–N(4)-C(9)	128.731	H(13)–O(1)–C(10)–H(17)	38.686
O(3)–N(6)	1.282	C(8)–N(4)–C(9)	105.731	C(8)–N(4)–C(7)–C(10)	97.765
N(4)–C(7)	1.476	C(8)–N(5)–C(11)	106.488	C(8)–N(4)–C(7)–H(14)	−23.003
N(4)–C(8)	1.378	O(2)–N(6)–O(3)	123.24	C(8)–N(4)–C(7)–H(15)	−140.654
N(4)–C(9)	1.402	O(2)–N(6)–C(9)	117.515	C(9)–N(4)–C(7)–C(10)	−81.667
N(5)–C(8)	1.348	O(3)–N(6)–C(9)	119.244	C(9)–N(4)–C(7)–H(14)	157.565
N(5)–C(11)	1.37	N(4)–C(7)–C(10)	112.756	C(9)–N(4)–C(7)–H(15)	39.913
N(6)–C(9)	1.411	N(4)–C(7)–H(14)	107.809	C(7)–N(4)–C(8)–N(5)	−179.647
C(7)–C(10)	1.534	N(4)–C(7)–H(15)	108.449	C(7)–N(4)–C(8)–C(12)	1.413
C(7)–H(14)	1.087	C(10)–C(7)–H(14)	109.32	C(9)–N(4)–C(8)–N(5)	−0.107
C(7)–H(15)	1.084	C(10)–C(7)–H(15)	109.615	C(9)–N(4)–C(8)–C(12)	−179.047
C(8)–C(12)	1.486	H(14)–C(7)–H(15)	108.805	C(7)–N(4)–C(9)–N(6)	−0.255
C(9)–C(11)	1.384	N(4)–C(8)–N(5)	111.32	C(7)–N(4)–C(9)–C(11)	179.725
C(10)–H(16)	1.088	N(4)–C(8)–C(12)	124.628	C(8)–N(4)–C(9)–N(6)	−179.775
C(10)–H(17)	1.089	N(5)–C(8)–C(12)	124.044	C(8)–N(4)–C(9)–C(11)	0.205
C(11)–H(18)	1.072	N(4)–C(9)–N(6)	125.362	C(11)–N(5)–C(8)–N(4)	−0.036
C(12)–H(19)	1.093	N(4)–C(9)–C(11)	106.904	C(11)–N(5)–C(8)–C(12)	178.912
C(12)–H(20)	1.087	N(6)–C(9)–C(11)	127.734	C(8)–N(5)–C(11)–C(9)	0.169
C(12)–H(21)	1.093	O(1)–C(10)–C(7)	109.19	C(8)–N(5)–C(11)–H(18)	179.827
		O(1)–C(10)–H(16)	105.954	O(2)–N(6)–C(9)–N(4)	−176.356
		O(1)–C(10)–H(17)	111.811	O(2)–N(6)–C(9)–C(11)	3.668
		C(7)–C(10)–H(16)	110.605	O(3)–N(6)–C(9)–N(4)	3.906
		C(7)–C(10)–H(17)	109.908	O(3)–N(6)–C(9)–C(11)	−176.07
		H(16)–C(10)–H(17)	109.314	N(4)–C(7)–C(10)–O(1)	−177.424
		N(5)–C(11)–C(9)	109.557	N(4)–C(7)–C(10)–H(16)	−61.207
		N(5)–C(11)–H(18)	122.773	N(4)–C(7)–C(10)–H(17)	59.589
		C(9)–C(11)–H(18)	127.669	H(14)–C(7)–C(10)–O(1)	−57.523
		C(8)–C(12)–H(19)	112.065	H(14)–C(7)–C(10)–H(16)	58.694
		C(8)–C(12)–H(20)	107.873	H(14)–C(7)–C(10)–H(17)	179.49
		C(8)–C(12)–H(21)	112.479	H(15)–C(7)–C(10)–O(1)	61.658
		H(19)–C(12)–H(20)	108.142	H(15)–C(7)–C(10)–H(16)	177.876
		H(19)–C(12)–H(21)	107.708	H(15)–C(7)–C(10)–H(17)	−61.329
		H(20)–C(12)–H(21)	108.438	N(4)–C(8)–C(12)–H(19)	63.444
				N(4)–C(8)–C(12)–H(20)	−177.627
				N(4)–C(8)–C(12)–H(21)	−58.084
				N(5)–C(8)–C(12)–H(19)	−115.365
				N(5)–C(8)–C(12)–H(20)	3.565
				N(5)–C(8)–C(12)–H(21)	123.107
				N(4)–C(9)–C(11)–N(5)	−0.235
				N(4)–C(9)–C(11)–H(18)	−179.872
				N(6)–C(9)–C(11)–N(5)	179.744
				N(6)–C(9)–C(11)–H(18)	0.107

Table 5 provides the optimized geometric parameters of metronidazole in water at 298.15 K. The optimized parameters in water reveal significant alterations; for instance, the O(1)–H(13) bond shortens to 0.964 Å and there are changes in angles, such as C(7)–N(4)–C(9) changing to 129.398°, suggesting that solvent interactions promote changes in molecular geometry, potentially enhancing hydrogen bonding and affecting overall stability. Moreover, the dihedral angle H(15)–C(7)–C(10)–H(16) changes from 177.876° in the gas phase (Table 4) to 179.14° in water (Table 5). This slight increase indicates a solvent-induced conformational adjustment. The polar water environment stabilizes a more extended geometry, reflecting the influence of solvation on molecular structure.

**Table 5:** Calculated optimized parameters of metronidazole in water at 298.15 K

Bond lengths	Values (Å)	Bond angles	Values (°)	Dihedral angles	Values (°)
O(1)–C(10)	1.425	C(10)–O(1)–H(13)	109.023	H(13)–O(1)–C(10)–C(7)	−75.819
O(1)–H(13)	0.964	C(7)–N(4)–C(8)	125.241	H(13)–O(1)–C(10)–H(16)	164.772
O(2)–N(6)	1.236	C(7)–N(4)–C(9)	129.398	H(13)–O(1)–C(10)–H(17)	46.848
O(3)–N(6)	1.237	C(8)–N(4)–C(9)	105.349	C(8)–N(4)–C(7)–C(10)	97.426
N(4)–C(7)	1.472	C(8)–N(5)–C(11)	106.317	C(8)–N(4)–C(7)–H(14)	−23.158
N(4)–C(8)	1.36	O(2)–N(6)–O(3)	123.393	C(8)–N(4)–C(7)–H(15)	−140.198
N(4)–C(9)	1.393	O(2)–N(6)–C(9)	117.285	C(9)–N(4)–C(7)–C(10)	−81.203
N(5)–C(8)	1.338	O(3)–N(6)–C(9)	119.322	C(9)–N(4)–C(7)–H(14)	158.212
N(5)–C(11)	1.35	N(4)–C(7)–C(10)	112.076	C(9)–N(4)–C(7)–H(15)	41.173
N(6)–C(9)	1.41	N(4)–C(7)–H(14)	107.324	C(7)–N(4)–C(8)–N(5)	−179.039
C(7)–C(10)	1.535	N(4)–C(7)–H(15)	108.547	C(7)–N(4)–C(8)–C(12)	1.878
C(7)–H(14)	1.089	C(10)–C(7)–H(14)	109.767	C(9)–N(4)–C(8)–N(5)	−0.137
C(7)–H(15)	1.088	C(10)–C(7)–H(15)	110.542	C(9)–N(4)–C(8)–C(12)	−179.22
C(8)–C(12)	1.489	H(14)–C(7)–H(15)	108.465	C(7)–N(4)–C(9)–N(6)	−1.483
C(9)–C(11)	1.382	N(4)–C(8)–N(5)	111.996	C(7)–N(4)–C(9)–C(11)	179.043
C(10)–H(16)	1.092	N(4)–C(8)–C(12)	124.096	C(8)–N(4)–C(9)–N(6)	179.679
C(10)–H(17)	1.093	N(5)–C(8)–C(12)	123.901	C(8)–N(4)–C(9)–C(11)	0.204
C(11)–H(18)	1.079	N(4)–C(9)–N(6)	125.933	C(11)–N(5)–C(8)–N(4)	0.012
C(12)–H(19)	1.094	N(4)–C(9)–C(11)	106.735	C(11)–N(5)–C(8)–C(12)	179.097
C(12)–H(20)	1.089	N(6)–C(9)–C(11)	127.33	C(8)–N(5)–C(11)–C(9)	0.123
C(12)–H(21)	1.093	O(1)–C(10)–C(7)	110.431	C(8)–N(5)–C(11)–H(18)	179.997
		O(1)–C(10)–H(16)	106.426	O(2)–N(6)–C(9)–N(4)	−178.382
		O(1)–C(10)–H(17)	111.221	O(2)–N(6)–C(9)–C(11)	0.985
		C(7)–C(10)–H(16)	110.038	O(3)–N(6)–C(9)–N(4)	1.715
		C(7)–C(10)–H(17)	110.191	O(3)–N(6)–C(9)–C(11)	−178.918
		H(16)–C(10)–H(17)	108.445	N(4)–C(7)–C(10)–O(1)	−176.825
		N(5)–C(11)–C(9)	109.602	N(4)–C(7)–C(10)–H(16)	−59.627
		N(5)–C(11)–H(18)	123.218	N(4)–C(7)–C(10)–H(17)	59.908
		C(9)–C(11)–H(18)	127.18	H(14)–C(7)–C(10)–O(1)	−57.667
		C(8)–C(12)–H(19)	111.315	H(14)–C(7)–C(10)–H(16)	59.531
		C(8)–C(12)–H(20)	108.389	H(14)–C(7)–C(10)–H(17)	179.066
		C(8)–C(12)–H(21)	111.962	H(15)–C(7)–C(10)–O(1)	61.942
		H(19)–C(12)–H(20)	108.456	H(15)–C(7)–C(10)–H(16)	179.14
		H(19)–C(12)–H(21)	107.97	H(15)–C(7)–C(10)–H(17)	−61.325
		H(20)–C(12)–H(21)	108.661	N(4)–C(8)–C(12)–H(19)	64.067
				N(4)–C(8)–C(12)–H(20)	−176.735
				N(4)–C(8)–C(12)–H(21)	−56.892
				N(5)–C(8)–C(12)–H(19)	−114.909
				N(5)–C(8)–C(12)–H(20)	4.289
				N(5)–C(8)–C(12)–H(21)	124.132
				N(4)–C(9)–C(11)–N(5)	−0.207
				N(4)–C(9)–C(11)–H(18)	179.925
				N(6)–C(9)–C(11)–N(5)	−179.671
				N(6)–C(9)–C(11)–H(18)	0.46

### 3.2 Fourier transform infrared analysis

**Fig. 5:**
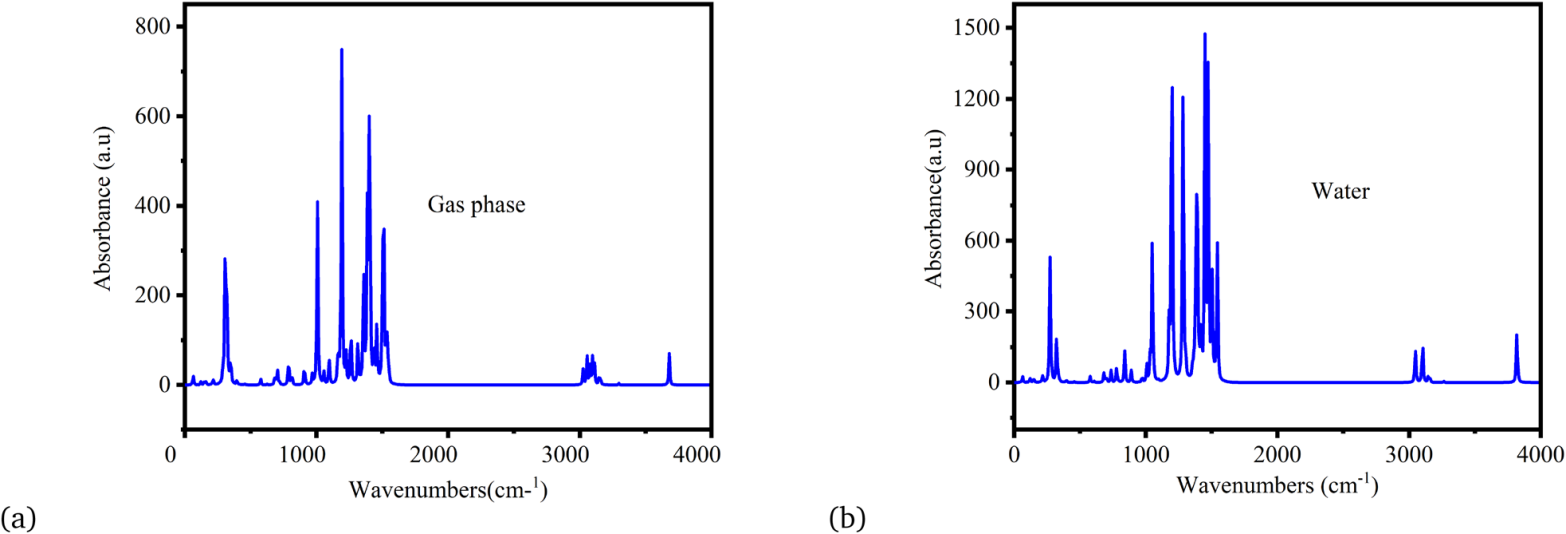
FTIR spectra of metronidazole: (a) gas phase and (b) water.

Fig. 5(a) and (b) provide the FTIR analysis of metronidazole in the vacuum and aqueous phases, respectively. The FTIR spectrum of metronidazole in the gas phase (Fig. 5(a)) presents a clear absorbance profile, providing insight into the molecule’s intrinsic vibrational modes, relatively free from intermolecular interactions. Prominent features include a strong and complex set of peaks observed particularly in the 1200–1600 cm^−1^ region. These absorptions are typically attributed to the stretching vibrations of the imidazole ring, encompassing C=C and C=N stretches, as well as contributions from the nitro group’s N–O stretching modes. The presence of smaller peaks below 1000 cm^−1^ signifies various bending and skeletal vibrations characteristic of the molecule’s unique structure. Furthermore, the very weak peaks observed around 3000 cm^−1^ are consistent with C–H stretching vibrations from the molecule. When compared to typical experimental FTIR spectra of organic molecules, this gas-phase spectrum aligns with expectations for a compound containing aromatic or heterocyclic rings and nitro groups, where strong absorptions in these regions are common. The sharpness of the peaks, characteristic of a gas-phase spectrum, further confirms the minimal broadening due to intermolecular collisions.^46^

Fig. 5(b), presenting the FTIR spectrum of metronidazole in water, vividly illustrates the profound impact of solvation on its vibrational modes, contrasting sharply with the gas-phase spectrum (Fig. 5(a)) and providing critical data for validating computational models.

### 3.3 Thermodynamic properties and dipole moment

Table 6 presents the thermodynamic properties and dipole moments of the metronidazole molecule calculated for both gas and various solvent conditions. The thermodynamic energy (*E*), expressed in kcal mol^−1^, is less in the water solvent (109.236 kcal mol^−1^) than in the gas phase (109.353 kcal mol^−1^), indicating that metronidazole shows n decrease in energy in more polar environments, likely due to stronger solute–solvent interactions and solvation effects. The heat capacity (*C*_V_) changes from 41.739 (gas phase) to 41.781 (water) cal mol^−1^ K^−1^, which suggests that the molecular vibrations and degrees of freedom change with solvent polarity. The dipole moments also show a significant increase from 3.588 D in the gas phase to 5.035 D in water, reflecting the enhanced polar character of metronidazole as solvation occurs, potentially influencing its biological activity and interactions with other polar molecules.

**Table 6:** The thermodynamic properties and dipole moments of metronidazole in gas and various solvents

Solvent	Thermodynamic properties			Dipole moment (D)			Total
	*E* (kcal mol^−1^)	*C*_V_ (cal mol^−1^ K^−1^)	*S* (cal mol^−1^ K^−1^)	*X*	*Y*	*Z*	
Gas	109.353	41.739	107.881	−2.841	2.019	0.853	3.588
Heptane	109.334	41.738	107.814	−3.193	2.213	0.948	3.999
Benzene	109.331	41.733	107.747	−3.272	2.251	0.971	4.088
Chloroform	109.305	41.740	107.684	−3.787	2.369	1.058	4.590
Dichloromethane	109.279	41.752	107.726	−3.956	2.467	1.127	4.796
Acetone	109.254	41.767	107.817	−4.074	2.541	1.178	4.944
Ethanol	109.250	41.770	107.837	−4.091	2.552	1.185	4.965
Methanol	109.245	41.774	107.862	−4.110	2.564	1.193	4.989
Acetonitrile	109.244	41.775	107.869	−4.115	2.568	1.195	4.996
Dimethyl sulfoxide	109.240	41.778	107.888	−4.129	2.577	1.201	5.013
Water	109.236	41.781	107.913	−4.146	2.588	1.208	5.035

Table 6 also provides the dipole moment values (*X*, *Y* and *Z* components and total) for the metronidazole molecule calculated in various solvents and the gas phase. The total dipole moment increases from 3.588 D in the gas phase to 5.035 D in water, indicating that the molecule gains significant polar character in more polar environments, which enhances its ability to interact with other polar molecules. The breakdown of the dipole moments into their *X*, *Y*, and *Z* components reveals that the increases in the total dipole moment are driven primarily by the *Y* and *Z* components, which shift from 2.019 D and 0.853 D in the gas phase to 2.588 D and 1.208 D in water, respectively. This suggests that solvent polarity particularly affects the molecular orientation and distribution of charge within metronidazole. Moreover, the negative *X* component suggests a conventional orientation of dipole moments, possibly reflecting the structural asymmetry of the molecule. Overall, the increase in dipole moment with increasing solvent polarity highlights how solvation can increase the molecular polarity of metronidazole, impacting its solubility and reactivity in biological systems, thereby playing a crucial role in its pharmacological behavior.

### 3.4 HOMO–LUMO analysis

Table 7 presents the HOMO, LUMO, energy gap, and chemical reactivity for the metronidazole molecule. The HOMO values range from −3.238 eV in the gas phase to −2.919 eV in the gas phase to −3.023 eV in water (a high-polarity solvent). This suggests that the electron-donating ability of metronidazole decreases as the solvent polarity increases. The increase in the HOMO energy level indicates greater stability in polar environments. The LUMO values remain relatively consistent, ranging from −7.399 eV (gas phase) to −7.283 eV (water phase), reflecting a minor influence of the solvent on the electron-accepting capability of the molecule.

**Table 7:** Calculations of frontier molecular orbital (FMO) parameters (eV) and chemical reactivity parameters for the metronidazole molecule

Solvent polarity	FMO parameters (eV)			Chemical reactivity parameters				
	HOMO	LUMO	Δ*E*	*μ*_cp_	*η*	*χ*	*S*_g_	*ω*
Gas	−2.919	−7.399	4.471	5.159	2.240	−5.159	0.212	5.634
Heptane	−2.955	−7.349	4.394	5.151	2.196	−5.151	0.218	5.791
Benzene	−2.964	−7.34	4.376	5.152	2.188	−5.152	0.219	5.825
Chloroform	−2.993	−7.314	4.321	5.154	2.1605	−5.1535	0.224	5.941
Dichloromethane	−3.008	−7.3	4.292	5.154	2.146	−5.154	0.226	6.002
Acetone	−3.017	−7.29	4.272	5.154	2.1365	−5.1535	0.227	6.04
Ethanol	−3.019	−7.288	4.269	5.154	2.1345	−5.1535	0.228	6.049
Methanol	−3.02	−7.286	4.266	5.153	2.133	−5.153	0.228	6.054
Acetonitrile	−3.021	−7.286	4.265	5.154	2.1325	−5.1535	0.228	6.057
Dimethyl sulfoxide	−3.022	−7.285	4.263	5.154	2.1315	−5.1535	0.228	6.061
Water	−3.023	−7.283	4.26	5.382	2.144	−5.382	0.226	6.554

**Fig. 6:**
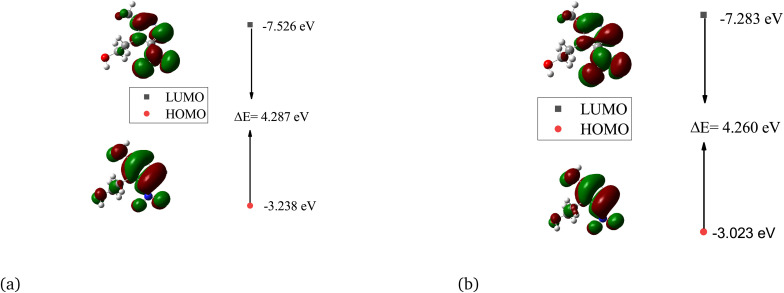
HOMO, LUMO, and HOMO–LUMO gaps for metronidazole in gas (a) and water (b).

Furthermore, Fig. 6 presents the molecular orbital surfaces for metronidazole, illustrating the HOMO, LUMO, and HOMO–LUMO gaps in both the gas phase and water. In the gas phase (Fig. 6(a)), the HOMO–LUMO gap is calculated to be 4.471 eV, indicating a relatively stable electronic configuration with significant electron density localized around the nitro and imidazole groups. This configuration supports the electron-donating ability of metronidazole, which is crucial for its reactivity and interaction with biological targets. In water (Fig. 6(b)), the HOMO–LUMO gap decreases slightly to 4.260 eV, suggesting that the presence of the polar solvent stabilizes the HOMO and increases the energy of the LUMO, facilitating easier electronic transitions. This decrease in the energy gap enhances the reactivity of metronidazole, increasing its susceptibility to electron transfer processes, which can facilitate its interaction with bacterial targets. The ability of metronidazole to dynamically adjust its electronic properties in response to the solvent environment may enhance its antibacterial activity, indicating that the effectiveness of the drug can be optimized in biological systems where polar environments are prevalent.

### 3.5 Density of states

**Fig. 7:**
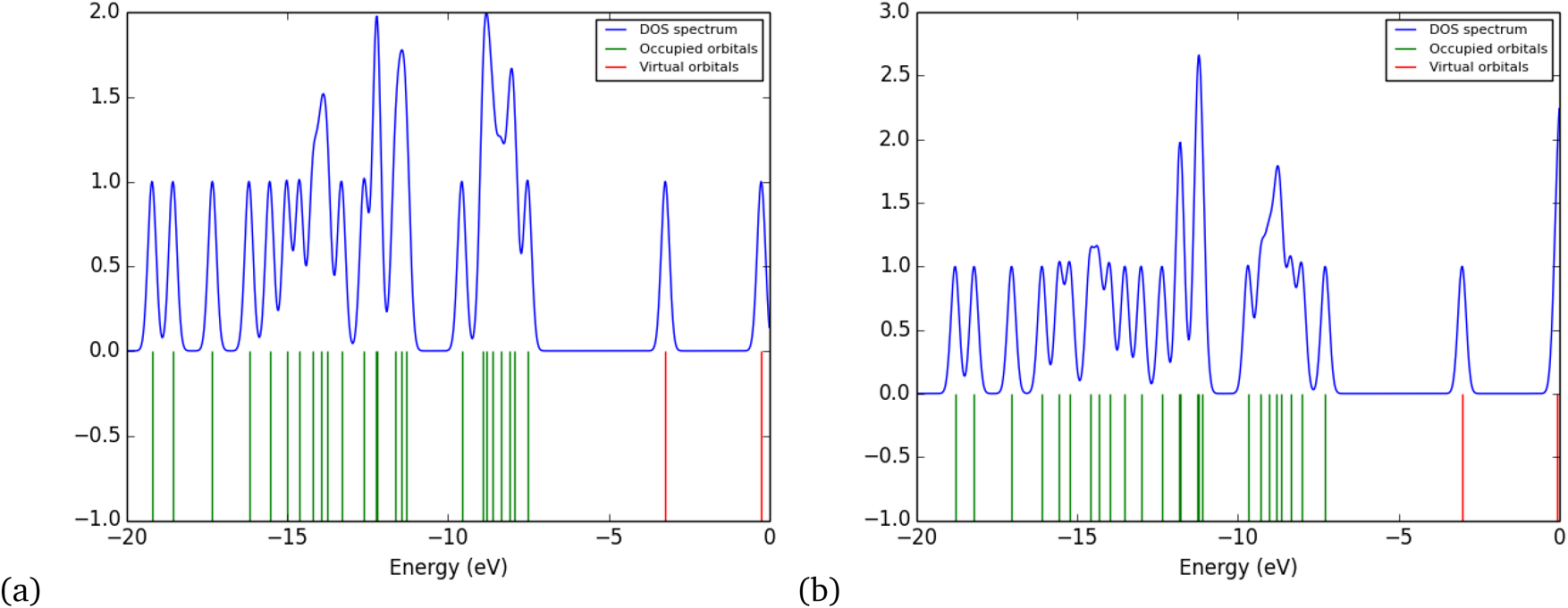
Density of states for metronidazole in a vacuum (a) and water (b).

The density of states (DOS) refers to the number of electronic states at each energy level that are available to be occupied by electrons in the molecule.^47^Fig. 7 shows the DOS of metronidazole. In the vacuum state (Fig. 7(a)), the DOS reveals distinct peaks associated with the energy levels of molecular orbitals, especially the highest occupied molecular orbital (HOMO) and the lowest unoccupied molecular orbital (LUMO). These peaks indicate the positions of energetic states that can participate in chemical reactions, providing insight into the reactivity and stability of metronidazole. The clear delineation of states in this environment suggests that the molecule maintains a well-defined electronic structure, allowing for predictable interactions on the basis of its inherent chemical properties. The DOS for metronidazole in water (Fig. 7(b)) shows broadening and shifts in the energy peaks, reflecting the influence of solvation on the molecular electronic environment. The interaction with water molecules likely leads to modifications in electronic states, such as stabilization of specific orbitals due to hydrogen bonding. This alteration can shift the HOMO/LUMO gap, which may enhance or diminish the molecule’s reactivity and interaction capabilities in a biological context. Moreover, the decreased intensity of some peaks in the water spectrum compared with that in the vacuum spectrum indicates that solvation contributes to reducing the availability of certain electronic states, potentially affecting the ability of a molecule to engage in interactions.^48^

### 3.6 Chemical reactivity

Chemical reactivity descriptors are essential parameters used to analyze the behavior and interactions of molecules in various chemical reactions.^49^ The ionization potential (IP) refers to the energy required to remove an electron from a neutral atom or molecule.^50^ Electron affinity (EA) denotes the energy change associated with the addition of an electron to a neutral atom or molecule.^51^ Chemical hardness (*η*) quantifies a molecule’s resistance to charge transfer and is defined as the second derivative of energy with respect to the number of electrons.^52^ Electronegativity (*χ*) describes an atom’s capacity to attract electrons within a chemical bond,^53^ whereas global softness (*S*_g_)^54^ acts as the inverse of chemical hardness, indicating how readily a molecule can accommodate additional charge. The global electrophilicity index (*ω*) measures a species’ ability to accept electrons, thus reflecting its reactivity toward nucleophiles. Collectively, these descriptors offer valuable insights into the stability, reactivity, and overall chemical behavior of compounds.^55^

Chemical reactivity is closely tied to the properties of frontier molecular orbitals, as follows:

1 IP = −*E*_HOMO_

2 EA = −*E*_LUMO_

Furthermore, the chemical reactivity (eqn (3)–(7)) was calculated according to Koopman’s theory^56^ as follows:

Chemical potential^57^3
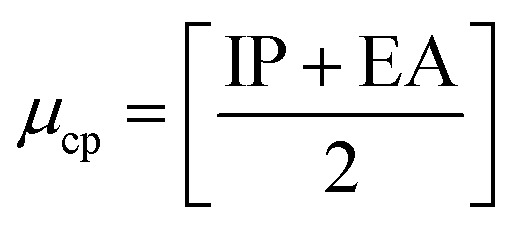


Chemical hardness^52^4
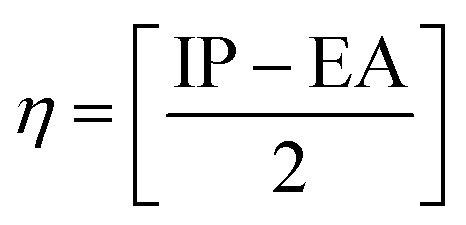


Electronegativity^53^5
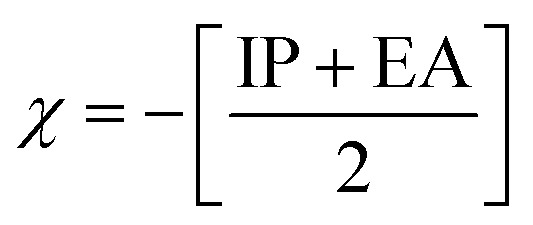


Global softness^58^6
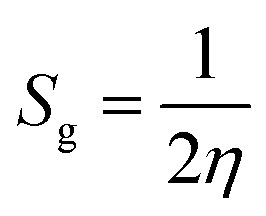


Global electrophilicity index^55^7
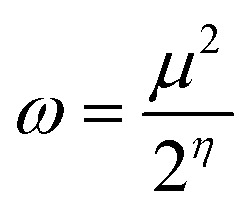


Table 7 also presents the chemical reactivity of metronidazole in both the gas phase and the solvent environment, as determined *via*eqn (1)–(7). The dipole moment (*μ*) values, ranging from 5.152 D in benzene to 5.382 D in the gas phase, suggest that metronidazole has a considerable permanent dipole, indicating a strong polar character that may affect its interactions in biological systems and enhance solubility in polar solvents. The chemical hardness (*η*) values of metronidazole, ranging from 2.240 eV in gas phase to 2.144 eV in water, suggest increased stability and reduced charge-transfer reactivity in polar solvents. The electronegativity (*χ*) values range from −5.152 to −5.382, which imply a moderate tendency for electron attraction, enhancing its potential reactivity with electrophiles. The softness (*S*_g_) values, which increase slightly with the reduction in chemical hardness, indicate that in more polar solvents, metronidazole is generally more reactive, suggesting that solvent effects could facilitate various chemical interactions. The global electrophilicity index (*ω*) increases from 5.634 eV in the gas phase to 6.554 eV in water. This rise indicates that metronidazole becomes a stronger electrophile in polar environments. It suggests enhanced ability to accept electrons, reflecting greater chemical reactivity in solution. Collectively, these parameters reveal how solvent polarity influences the chemical landscape of metronidazole, providing crucial insights into its reactivity and interactions in medicinal chemistry.

### 3.7 Electrostatic potential analysis

**Fig. 8:**
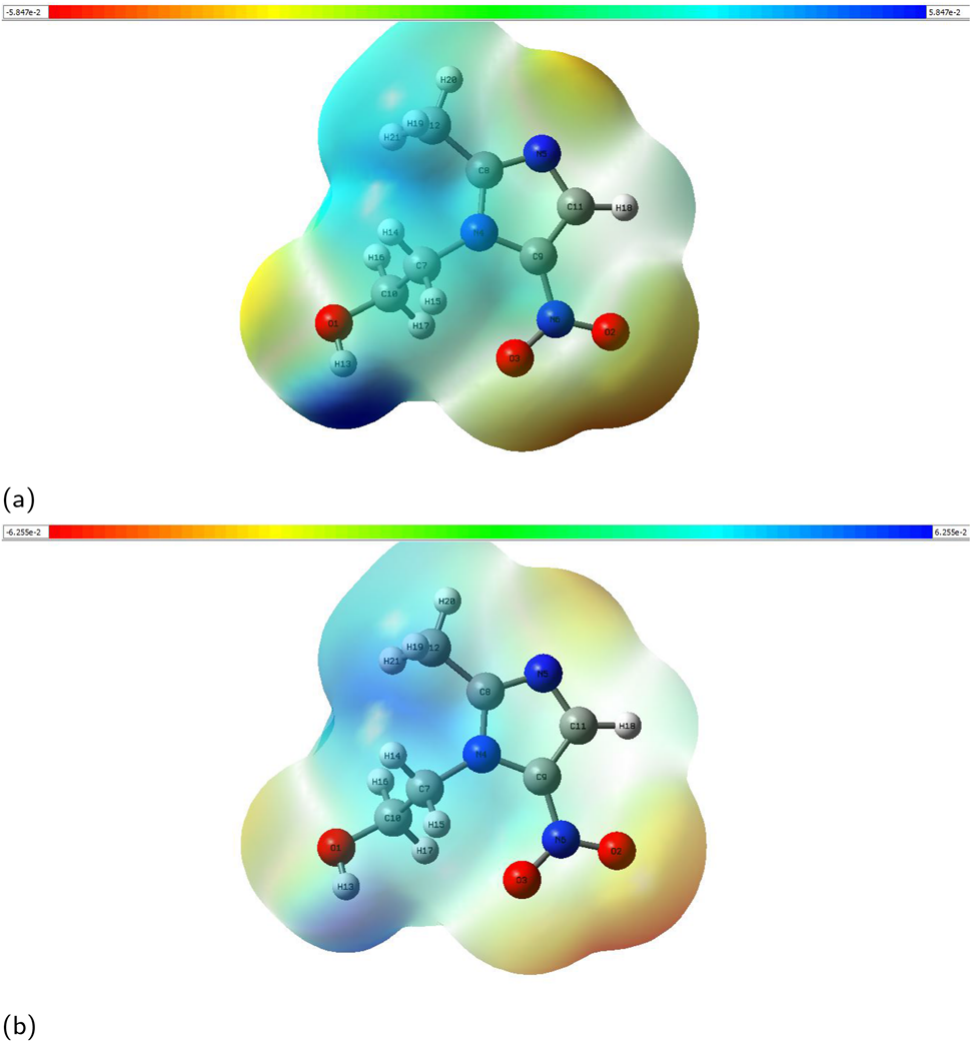
Electrostatic potential map (ESP) of metronidazole calculated in (a) a vacuum and (b) water.

Electrostatic potential (ESP) maps the charge distribution of a molecule, showing regions of electrophilic and nucleophilic reactivity.^59^Fig. 8 shows the electrostatic potential (ESP) maps of metronidazole in (a) a vacuum and (b) water. The color scale ranges from red (most negative ESP) to blue (most positive ESP). In a vacuum (Fig. 8(b)), the ESP varies from approximately −4.25 × 10^−2^ to +1.85 × 10^−2^ a.u. In water (Fig. 8(a)), it ranges from −4.25 × 10^−2^ to +1.52 × 10^−2^ a.u. This shows a reduction of 0.33 × 10^−2^ a.u. in the maximum ESP due to solvation. The red-orange regions around the nitro group (–NO_2_) and hydroxyl group (–OH) represent electron-rich zones. These are likely sites for electrophilic attack. The blue regions near hydrogen atoms (on the imidazole ring and –CH_2_ groups) indicate electron-deficient areas. These are potential sites for nucleophilic attack. In water, the ESP becomes more uniform. This implies stabilization of polar functional groups by the solvent. The maps highlight reactive sites and the solvent effect on charge distribution.

### 3.8 UV–VIS spectrum

**Fig. 9:**
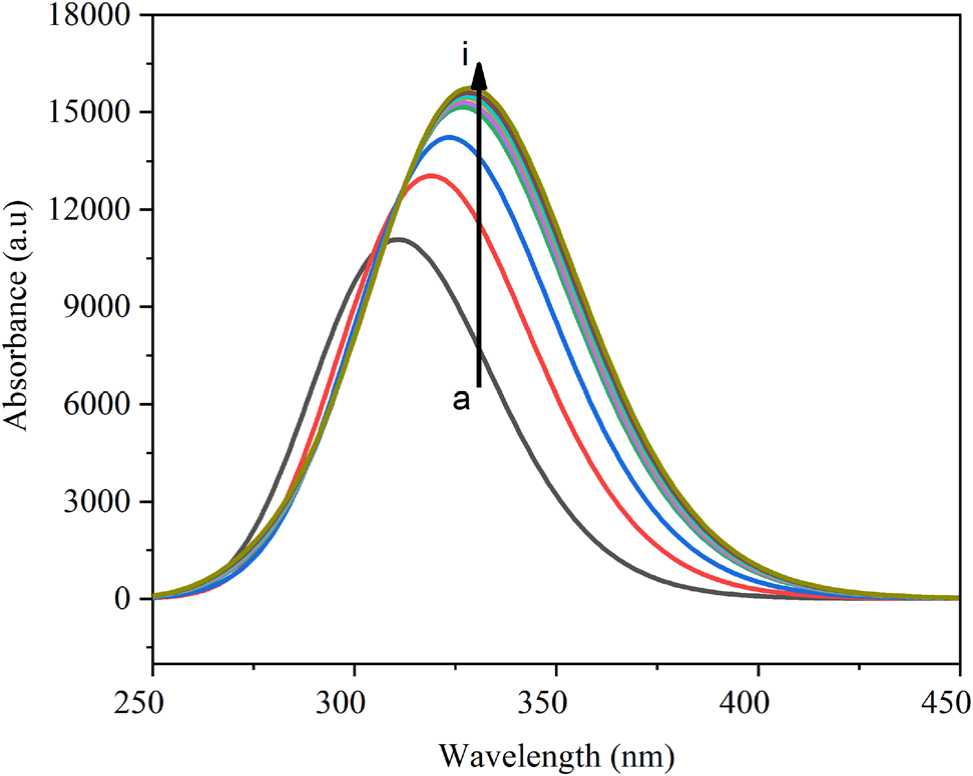
UV–Vis absorption spectra of metronidazole in various solvents: (a): benzene; (b): chloroform; (c): dichloromethane; (d): acetone; (e): ethanol; (f): methanol; (g): acetonitrile; (h): dimethyl sulfoxide; and (i): water.

Fig. 9 shows the UV-Vis absorption spectra of metronidazole in various solvents. The UV-Vis absorption spectra of metronidazole exhibit a single, prominent absorption band consistently across all investigated solvents, indicating a characteristic electronic transition of the molecule. The *λ*_max_ values, detailed in Table 8, show a clear bathochromic shift (red-shift) with increasing solvent polarity, ranging from 324 nm in non-polar benzene to 329.87 nm in highly polar water. Concurrently, the oscillator strengths (*f*) generally increase with solvent polarity, suggesting a more allowed and intense transition in polar environments. This collective behavior underscores a significant solvatochromic effect on metronidazole’s electronic excited states. Changes in absorption spectra due to solute–solvent interactions are vital for antibacterial activity, as they influence bioavailability and the ability to penetrate bacterial membranes.^60^

**Table 8:** Maximum absorption wavelengths (*λ*_max_), oscillator strengths, and assigned electronic transitions in various solvents, from TD-DFT calculations

Solvent	*λ*_max_ (nm)	Oscillator strength (*f*)	Assigned transition
Benzene	324	0.0003	π → π∗ (HOMO → LUMO)
Chloroform	324.15	0.0043	*n* → π∗ (HOMO-1 → LUMO)
DCM	324.81	0.1657	Intramolecular charge transfer (HOMO → LUMO)
Acetone	327.73	0.3492	π → π∗ (HOMO → LUMO)
Ethanol	328.22	0.3555	*n* → π∗ (HOMO-2 → LUMO)
Methanol	328.78	0.3616	Intramolecular charge transfer (HOMO → LUMO)
Acetonitrile	328.94	0.3632	π → π∗ (HOMO → LUMO+1)
DMSO	329.34	0.367	*n* → π∗ (HOMO-1 → LUMO)
Water	329.87	0.3716	Intramolecular charge transfer (HOMO → LUMO)

Table 8 shows the electronic transitions derived from TD-DFT calculations, which reveal a complex interplay between the molecule and its solvent environment. π → π∗ and *n* → π∗ transitions are identified, and a dominant feature appears to be intramolecular charge transfer (ICT) in several polar solvents like DCM, methanol, and water. The observed red-shift in *λ*_max_ and increased oscillator strengths for these transitions in polar solvents are consistent with the stabilization of a more polar excited state relative to the ground state. This highlights the sensitivity of metronidazole’s electronic structure to solvent polarity, significantly influencing its absorption characteristics.

### 3.9 Molecular docking studies

#### 3.9.1 Ligand–protein interactions

The receptor–ligand interactions of metronidazole with selected antibacterial proteins were analyzed *via* AutoDock Vina to determine the binding affinity and RMSD, as shown in Table 9. The binding affinity, measured in kcal mol^−1^, indicates the strength of the interaction, with more negative values suggesting stronger binding. The metronidazole–4kov complex has the strongest binding affinity of −5.3 kcal mol^−1^, indicating a strong interaction, but its RMSD values (1.721 for the lower bound and 2.304 for the upper bound) suggest that the docking pose is less accurate. In comparison, the metronidazole–8fb0 complex shows a slightly weaker affinity of −4.3 kcal mol^−1^ but has the most accurate pose, with RMSD values of 1.203 (lower bound) and 2.014 (upper bound). The other complexes, including metronidazole–3q5p (−4.5 kcal mol^−1^), display moderate affinities but with progressively higher RMSD values, indicating that while the binding is still favorable, the docking poses are less precise. The lowest binding affinity and RMSD (<2.5 Å) indicate the strongest interaction between the drug and the target protein, suggesting optimal conditions for antibacterial activity.^61^

**Table 9:** Binding affinities and RMSD values for metronidazole with selected antibacterial proteins

Ligand	Protein	Affinity (kcal mol^−1^)	RMSD (l.b)	RMSD (u.b)
Metronidazole	8fb0	−4.3	1.203	2.014
Metronidazole	4kov	−5.3	1.721	2.304
Metronidazole	5j62	−4.8	1.815	2.139
Metronidazole	3q5p	−4.5	1.929	2.206

Table 10 shows the interactions between metronidazole and four antibacterial protein targets. These interactions involve amino acid residues such as ARG45, HIS97, TYR68, and PHE89, which contribute to stabilizing the ligand–protein complexes. Notably, conventional hydrogen bonds were observed with bond lengths typically ranging between 2.0–3.0 Å, suggesting strong binding affinities, while interactions like π–π stacking and π–alkyl contacts indicate secondary stabilization. These findings emphasize both common (*e.g.*, hydrogen bonding) and less common (*e.g.*, amide–π stacking, π–σ) interactions, which are important for understanding ligand orientation, selectivity, and overall binding strength. These findings suggest that both hydrogen bonding and hydrophobic interactions are crucial for metronidazole’s effectiveness against bacterial targets.

**Table 10:** Nonbonding interactions between metronidazole and antibacterial protein targets

Ligand	Protein ID	Amino acid	Distance	Category	Types
Metronidazole	8fb0	ARG45	2.209	Hydrogen bond	Conventional hydrogen bond
		ARG45	3.193	Hydrogen bond	Conventional hydrogen bond
		HIS97	2.850	Hydrogen bond	Conventional hydrogen bond
		ASP44	2.062	Hydrogen bond	Conventional hydrogen bond
		ASP44	4.369	Hydrophobic	Amide-pi stacked
		ARG45	5.278	Hydrophobic	Pi-alkyl
Metronidazole	4kov	TYR68	2.882	Hydrogen bond	Conventional hydrogen bond
		ASN80	2.058	Hydrogen bond	Conventional hydrogen bond
		CYS29	2.694	Hydrogen bond	Conventional hydrogen bond
		CYS29	2.703	Hydrogen bond	Conventional hydrogen bond
		GLY79	3.583	Hydrogen bond	Carbon hydrogen bond
		SER116	3.691	Hydrogen bond	Carbon hydrogen bond
		TYR68	3.290	Hydrogen bond	Pi-donor hydrogen bond
		TYR68	5.015	Hydrophobic	Pi–pi stacked
		PRO31	4.465	Hydrophobic	Pi-alkyl
Metronidazole	5j62	LEU88	2.98767	Hydrogen bond	Conventional hydrogen bond
		PHE89	2.19381	Hydrogen bond	Conventional hydrogen bond
		GLN60	2.00908	Hydrogen bond	Conventional hydrogen bond
		LYS87	3.55147	Hydrophobic	Pi-sigma
		LYS64	4.0364	Hydrophobic	Pi-alkyl
Metronidazole	3q5p	TYR152	2.57263	Hydrogen bond	Conventional hydrogen bond
		GLU253	2.59958	Hydrogen bond	Conventional hydrogen bond
		TAL148	2.13857	Hydrogen bond	Conventional hydrogen bond
		VAL147	3.55312	Hydrophobic	Pi-sigma

**Fig. 10:**
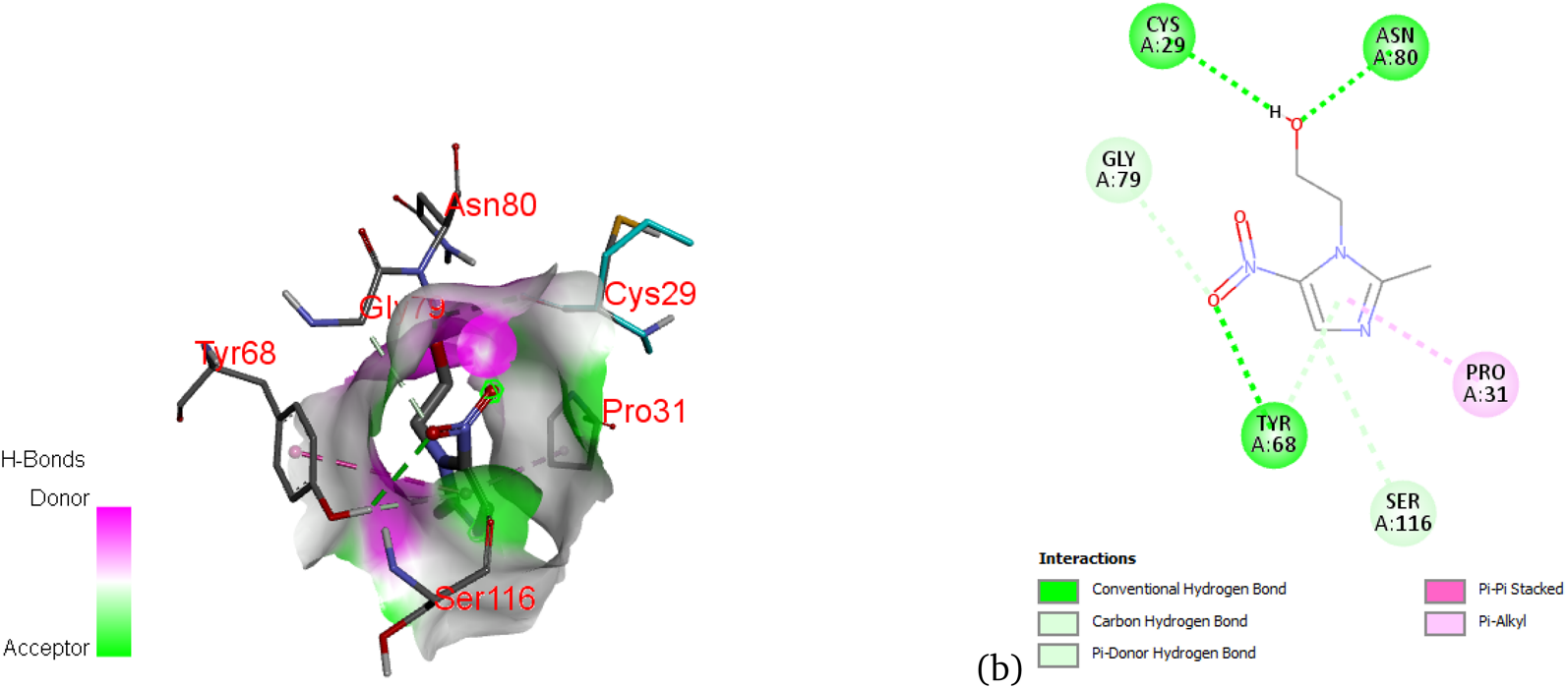
3D (a) and 2D (b) map of nonbonding interactions between the 8fb0 protein and metronidazole.

**Fig. 11:**
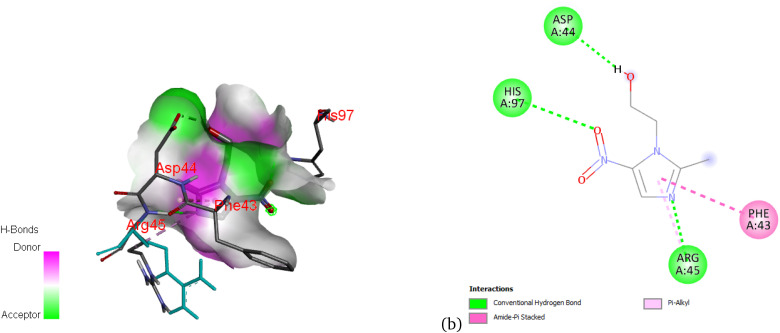
3D (a) and 2D (b) map of the nonbonding interactions between the 4kov protein and metronidazole.

**Fig. 12:**
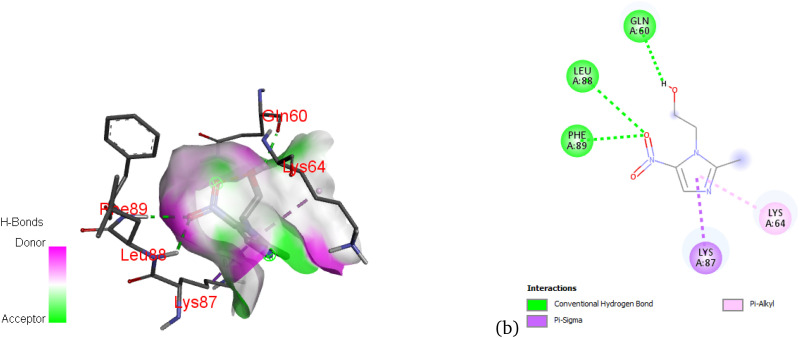
3D (a) and 2D (b) map of nonbonding interactions between the 5j62 protein and metronidazole.

**Fig. 13:**
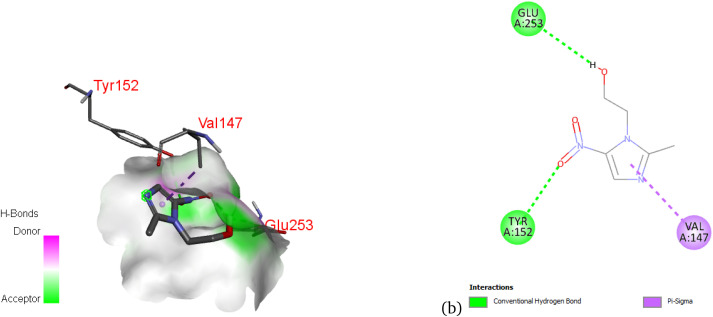
3D (a) and 2D (b) map of nonbonding interactions between the 3q5p protein and metronidazole.

Furthermore, Fig. 10–13 illustrate the nonbonding interactions between metronidazole and 8fb0, 4kov, 6ko5, and 3q5p, respectively.

As shown in Fig. 10, metronidazole interacts with the 8fb0 protein through a network of hydrogen bonds and hydrophobic contacts. Notably, conventional hydrogen bonds are observed with residues such as Ser116 and Cys29, while π-alkyl interactions are formed with Tyr68, Pro31, and Asn80, which likely contribute to the structural stability of the complex. The surface topology in the 3D map highlights regions where donor and acceptor interactions are spatially localized, supporting a favorable binding conformation.

Fig. 11 displays the interaction profile of metronidazole with the 4kov protein. Strong conventional hydrogen bonds are observed with residues Asp44, His97, and Arg45, suggesting these residues play a vital role in ligand recognition. Additionally, a π–alkyl interaction with Phe43 may reinforce ligand binding *via* van der Waals forces. The dense surface contact observed in the 3D image reflects a well-fitted binding pocket with high complementarity.

Fig. 12 presents the nonbonding interactions between metronidazole and the 5j62 protein. As shown, the ligand forms conventional hydrogen bonds with Gln60 and Leu88, and π-alkyl interactions with Lys64 and Lys87, which stabilize the complex. These interactions suggest that metronidazole is well-accommodated in the binding pocket, contributing to its binding affinity through hydrogen bonding and hydrophobic contacts.

Fig. 13 shows the critical nonbonding interactions between the 3q5p protein and metronidazole. The 3D representation (a) clearly depicts a strong hydrogen bond with Glutamine 253 (Glu253) and a stabilizing π–σ interaction with Valine 147 (Val147). The 2D diagram (b) reinforces these findings and reveals an additional conventional hydrogen bond with Tyrosine 152 (Tyr152). These precise molecular contacts collectively underscore a robust and specific binding affinity. This detailed mapping of interactions is essential for elucidating the drug’s mechanism and potential implications for its biological activity.

#### 3.9.2 Multiple ligands and antibacterial protein interactions

Table 11 presents the effects of combining metronidazole with different ligands on the binding affinities across four bacterial proteins (8fb0, 4kov, 5j62, and 3q5p). The binding affinities for combinations of metronidazole with secnidazole, tizoxanide, and caffeine are greater than the individual metronidazole values in Table 9. For instance, metronidazole + tizoxanide has a stronger binding affinity, with −8.0 kcal mol^−1^ for 8fb0 and −8.7 kcal mol^−1^ for 4kov, than metronidazole alone with 8fb0, which has a binding affinity of −4.3 kcal mol^−1^. The combination of all four ligands (metronidazole + secnidazole + tizoxanide + caffeine) achieves the highest affinity at −8.8 kcal mol^−1^ for 4kov. This trend suggests that combining ligands enhances the stability and strength of interactions, likely due to synergistic effects such as increased hydrogen bonding or hydrophobic interactions. Specifically, the inclusion of caffeine, a well-known modulator of protein–ligand interactions, aligns with previous research, such as that of Sherefedin *et al.*, which discussed the interaction of caffeine with hydroxycinnamic acids such as *p*-coumaric, caffeic, and ferulic acids.^25^ Similarly, Asemare *et al.* reported enhanced binding between caffeine and metformin hydrochloride when docking with AMP-activated protein kinase, further supporting the role of caffeine in strengthening molecular docking.^62^

**Table 11:** Binding affinities (kcal mol^−1^) of multiple ligand combinations with selected antibacterial proteins

Ligands	Protein			
	8fb0	4kov	5j62	3q5p
Metronidazole + secnidazole	−4.8	−6.8	−5.5	−5.6
Metronidazole + tizoxanide	−8.0	−8.7	−8.0	−7.1
Metronidazole + caffeine	−6.0	−6.7	−6.5	−6.1
Metronidazole + secnidazole + tizoxanide	−8.1	−8.7	−8.3	−7.1
Metronidazole + secnidazole + tizoxanide + caffeine	−8.2	−8.8	−8.1	−7.0

## 4 Conclusions

This work investigated the effects of solvent polarity on the structural, thermodynamic, and electronic properties of metronidazole *via* DFT and molecular docking analysis for antibacterial applications. This study highlights the significant influence of computational methods and environmental factors on key molecular properties, such as dipole moment, polarizability, and thermal energy. Under both vacuum and aqueous conditions, water enhances solvation properties, leading to an increased dipole moment and polarizability, which reflect stronger molecular interactions. Structural optimizations reveal that solvent interactions induce notable geometric alterations, potentially enhancing molecular stability and reactivity in biological systems. The HOMO–LUMO gap analysis suggests that polar solvents, particularly water, increase the reactivity, facilitating the electron transfer essential for antibacterial activity. DOS analysis indicated that solvation broadens and shifts energy peaks, further altering the molecule’s reactivity. Chemical reactivity descriptors, which are crucial for biological interactions, demonstrate enhanced stability, solubility, and bioavailability in polar environments. MEP maps and UV-Vis spectra confirmed that solvent polarity affects a molecule’s electrostatic potential and excited states, influencing its antibacterial efficacy. Molecular docking studies revealed that metronidazole strongly interacts with antibacterial proteins, especially in the 4kov complex, with a binding affinity of −5.3 kcal mol^−1^, although its RMSD values suggest less accuracy. In contrast, the metronidazole–8fb0 complex, with a binding affinity of −4.3 kcal mol^−1^, displays the most accurate docking pose. Nonbonding interactions, such as hydrogen bonds and hydrophobic contacts, contribute significantly to the binding affinity of metronidazole, particularly with amino acids such as ARG45, TYR68, and HIS97. Moreover, combining metronidazole with other ligands, such as secnidazole, tizoxanide, and caffeine, substantially improved the binding affinities, with the combination of all four ligands achieving the highest affinity of −8.8 kcal mol^−1^ for the 4kov protein.

## Author contributions

Desta Ragessa Golja (PhD) led the conceptualization, data preparation, simulations, analysis, and drafting of the initial manuscript. Megersa Olumana Dinka (Prof.) provided supervision and guidance and participated in the review process. Umer Sherefedin (PhD) conducted density functional theory (DFT) calculations *via* Gaussian 09 and performed molecular docking studies with AutoDock Vina and PyRx (Vina). Abebe Belay (Prof.) performed DFT calculations using Gaussian 09 software, carried out editing, reviewing, and supervising. Dereje Gelanu (PhD) contributed to the DFT simulation sections and assisted with the final manuscript revisions. Gadisa Deme Megersa conducted all molecular docking simulations using AutoDock Vina integrated with PyRx, writing the original draft and editing.

## Conflicts of interest

The authors affirm that they have no conflicts of interest to disclose.

## Data Availability

All relevant data supporting the findings of this study are included within the article.
